# Analysis and Synthesis of Traffic Scenes from Road Image Sequences

**DOI:** 10.3390/s20236939

**Published:** 2020-12-04

**Authors:** Sheng Yuan, Yuting Chen, Huihui Huo, Li Zhu

**Affiliations:** School of Software Engineering, Xi’an Jiaotong University, Xi’an 710049, China; ysheng@mail.xjtu.edu.cn (S.Y.); chenyuting@stu.xjtu.edu.cn (Y.C.); huohuihui@stu.xjtu.edu.cn (H.H.)

**Keywords:** traffic elements detection, road scene inpainting, road scene modeling, analysis and synthesis

## Abstract

Traffic scene construction and simulation has been a hot topic in the community of intelligent transportation systems. In this paper, we propose a novel framework for the analysis and synthesis of traffic elements from road image sequences. The proposed framework is composed of three stages: traffic elements detection, road scene inpainting, and road scene reconstruction. First, a new bidirectional single shot multi-box detector (BiSSD) method is designed with a global context attention mechanism for traffic elements detection. After the detection of traffic elements, an unsupervised CycleGAN is applied to inpaint the occlusion regions with optical flow. The high-quality inpainting images are then obtained by the proposed image inpainting algorithm. Finally, a traffic scene simulation method is developed by integrating the foreground and background elements of traffic scenes. The extensive experiments and comparisons demonstrate the effectiveness of the proposed framework.

## 1. Introduction

Traffic scene simulation and modeling has been a hot topic in the community of intelligent transportation systems. The analysis and synthesis of traffic scene is the foundation for traffic simulation and modeling. A wide range of applications has been developed based on scene analysis and synthesis, including the evaluation of unmanned vehicle algorithms [1], traffic scenes construction [2,3,4], and the advanced driver assistant systems (ADAS) [5]. The testing and evaluation of unmanned vehicle algorithms are crucially important for unmanned vehicles. There are basically two types for unmanned vehicle evaluation methods: field test and off-line test. As the traditional field test is unsafe and demands too much time cost, the off-line test of unmanned vehicles has become popular in recent years. Based on the analysis and synthesis of traffic scenes, the off-line test method is repeatable. Apart from unmanned vehicle evaluation, another application is to construct the 3D virtual traffic roads by combining background scene and foreground traffic elements. On the basis of the virtual traffic roads, a variety of advanced driver assistant systems have been developed.

In order to implement the analysis and synthesis of traffic scene, the framework proposed in this paper mainly consists of three parts: traffic elements detection, road scene inpainting, and road scene modeling. First of all, the traffic elements detection method separates the foreground objects from the background in traffic scenes. In real scenes, object detection not only requires real-time speed, but also needs high accuracy. In order to consider both detection speed and accuracy, we propose a bidirectional single shot multi-box detector (BiSSD) method, which combines single shot multi-box detector (SSD) [6] feature extraction and an improved bidirectional feature pyramid network (BiFPN) [7,8] feature fusion. The feature fusion is applied to SSD feature extraction. Based on BiFPN method, we modify the addition operation of BiFPN of the spatial level to the concatenate operation of channel level, so as to improve the efficiency of feature utilization. A feature fusion module is integrated after feature extraction, and a global context attention mechanism is added to the module. Moreover, the global context attention mechanism enables the model to make full use of the acquired features. Then, the image inpainting is applied to restore images after removing foreground objects from road image sequences. Image inpainting aims at restoring a pure image by filling in missing pixels. The previous works of image inpainting are categorized into two types: the traditional methods and deep learning methods. The traditional methods [9,10,11,12] are designed to find neighborhood pixels of missing region and fill them with adjacent pixels. This method requires prior information and does not incorporate context information of image sequences. In recent years, due to the rapid development of deep learning, more and more researchers apply convolutional neural networks (CNN) [13,14] and GAN [15] to the image inpainting. However, high-quality inpainting results need to feed plenty of training data, while the trained models are not robust enough. To overcome these drawbacks, we propose a novel method for jointly inpainting of optical flow and image content using an unsupervised CycleGAN [16] to ensure semantic coherence. Finally, the road scene modeling results can be applied to the synthesis of traffic scenes [17]. It can be utilized as an alternative method for real-world test of unmanned vehicles, thus saving a lot of testing time and energy. Based on the result of foreground modeling and background restoration, we then construct the road scene models from road image sequences.

The main contributions of this work can be summarized as follows.

Traffic elements detection: The feature fusion is integrated for SSD feature extraction. Based on the BiFPN method, we modify the addition operation of BiFPN in the spatial level of feature map to the concatenate operation of channel level, so as to improve the efficiency of the model in obtaining and utilizing feature information. An attention mechanism is applied to enable the model make full use of the desired features.

- Road scene inpainting: An unsupervised CycleGAN is developed to inpaint the missing region in optical flow which is generated from adjacent frames. The inconsistency between foreground and background optical flow information can be applied to restore the missing pixels of undesired regions. A Gaussian mixture model is adopted to further refine the undesired region.

- Road scene modeling: A novel road scene modeling method is developed using object detection and image inpainting, which can be applied to traffic scene simulation and evaluation.

The rest of the paper is organized as follows. Section 2 shows an overview of the related works. The proposed traffic elements detection method is presented in Section 3. In Section 4, the construction of the road scene models is introduced. The experiments and comparisons are shown in Section 5. Finally, we close this paper with conclusion and future works.

## 2. Related Works

The main purpose of this paper is to analyze and synthesize the traffic elements from road image sequences. The detection of traffic elements is an important precondition for road scene modeling. Thus, we put our work into context by discussing prior work in the fields of traffic elements detection and road scene modeling.

### 2.1. Traffic Elements Detection

The traffic elements mainly include the moving objects in the traffic scenes, such as pedestrians, cars and so on. Object detection is a basic task in the field of computer vision. Traditional object detection mainly relies on image features, such as SIFT [18], Haar [19,20], HOG [21,22], etc. The feature classification is then carried out by Adaboost [23], support vector machine (SVM) [24] and other classifiers. Because of the huge improvement in the image feature extraction capability brought about by convolutional neural network (CNN) models, such as VGG [25], ResNet [26], etc., the CNN model is applied to the field of object detection. According to whether it is necessary to generate proposals, the object detection methods based on CNN can be divided into two categories [27]: two-stage method and one-stage method. The typical two-stage method, represented by Faster R-CNN [28], is based on the core idea that the proposals are first generated, then the classifiers are utilized for regression and classification. The two-stage method shows high detection accuracy, but its speed performance is slow. One-stage methods, such as YOLO [29] and SSD [6], conduct intensive sampling of images at different scales and directly utilize the convolutional network for classification, which shows a relatively fast detection speed. Among the anchors learned through the one-stage network, only a few of them are beneficial to the parameters learning, which greatly affects the accuracy rate. In addition, each anchor mapped area needs to be input into the fully connected layer for classification and regression, which is time-consuming. The SSD method is superior to the YOLO method in both accuracy and speed. SSD adopts convolutional layers for detection and uses anchors of different scales to detect target objects of different sizes. In the object detection task, multi-scale feature fusion can effectively improve the performance of the model. The low-level features extracted from the shallow layers have higher resolution and less semantic information, and the high-level features extracted from deep layer contain rich semantic information, but lack the perception details. To utilize features of different levels, the multi-scale feature fusion combines the information of low-level and high-level features effectively. The fine-grained information is utilized, and thus the prediction accuracy of the small objects is improved. The existing feature fusion methods include FPN [7], PANet [30], NAS-FPN [31], BiFPN [8], etc. Feature Pyramid Network (FPN) is applied to conduct the upsampling operation for the high-level features. The low-level features are then combined to effectively percept the high-resolution and semantic information. Path Aggregation Network (PANet) focuses on the utilization of low-level features and introduces bottom-up path augmentation combined with adaptive feature pooling. The NAS-FPN method is optimized based on FPN, and uses the Neural Architecture Search (NAS) technology to design neural network structure of FPN automatically. The BiFPN method is improved from PANet by eliminating redundant edges and adding the skip connections mechanism. In this paper, we propose a method based on SSD feature extraction and improved BiFPN feature fusion method. The proposed method can achieve improvement in target frame regression accuracy, detection accuracy, and shows robust performance for small objects.

### 2.2. Road Scene Modeling

The road scene modeling process mainly consists of two stages: background inpainting and scene models construction. Image inpainting was first proposed as a general image processing problem that aims to recover the damaged or missing region of an image. The basic idea is simple: replace those missing regions with their neighboring pixels. A large amount of research is done for image inpainting. Nitzberg et al. [10] propose a technique to remove occlusion for image segmentation. Masnou et al. [11] propose a general variational formula to inpaint regions with simple topologies. Ballester et al. [12] introduce a new algorithm for still image inpainting. After the user selects the areas to restore, the algorithm automatically fills in with the surrounding pixels. These traditional methods perform well on simple structure but are very limited to complex objects, large missing area and non-repetitive texture. Recently, the neural networks are applied to image inpainting. Neural network models learn semantic information in the training data and are thus capable of generating realistic content that may not exist in the unmasked area. Xie et al. [14] were the first to train convolutional neural networks for image denoising and inpainting on small regions. Pathak et al. introduce the idea of adversarial loss from the generative adversarial network (GAN) [32] using a generator and discriminator. These methods utilize neural networks to achieve higher resolution and can deal with various types of irregular damage. Video inpainting is generally viewed as an extension of the image inpainting task with larger search space and temporally consistent constraints. At present, many video inpainting methods use patch-based algorithms. Wexler et al. [2] consider the video inpainting task as a global optimization problem where all missing portions could be filled in with patches from the available parts of the video with enforced global spatial-temporal consistency. They propose an iterative approach to solve the global optimization problem and yield magnificent results in an automatic way. By utilizing deep convolutional networks to inpaint undesired regions in videos, this approach achieves promising results. The method of Horry et al. [3] partitions an input image into regions of “left wall”, “right wall”, “back wall”, “ceiling” and “floor”. The foreground objects are assumed to stand perpendicularly to the floor plane. However, their method is only suitable for scenes with straight boundaries, which can not be applied to the curved road conditions. The method of Li et al. [1] constructs road scene models based on the road region detection results; however, the image inpainting is not incorporated to the scene modeling framework. In this paper, the proposed method divides into two stages. In the first stage, CycleGAN is applied to inpaint optical flow. In the second stage, the optical flow map after inpainting is applied to trace image content of corresponding background and obtain image sequences of pure backgrounds. Based on the results of road image inpainting, the traffic scenes can be constructed and simulated.

## 3. Traffic Elements Detection

### 3.1. Improved BiFPN Feature Fusion

**Figure 1:**
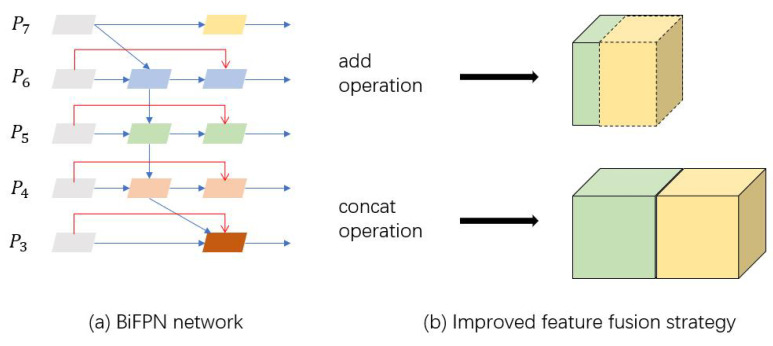
The flow diagram of the improved BiFPN method.

In this paper, SSD is adopted in the process of traffic elements detection, and the multi-scale feature maps are utilized to improve the detection results on objects of various sizes. The feature extraction process directly uses the convolutional layers to extract features of different scales. For the vanilla SSD network, feature fusion is not applied, thus a lot of fine-grained information is discarded in the deep layer of the network, and the ability to recognize small object of network is unsatisfactory. Therefore, after feature extraction in vanilla SSD, the improved BiFPN structure is appended for feature fusion. BiFPN eliminates redundant connection points in PANet and adds a skip connection mechanism similar to ResNet, which not only reduces computation, but also ensures the richness of fusion features. In this paper, an improved method based on BiFPN is proposed, as shown in Figure 1. The original BiFPN method adopts an add operation for feature fusion between the hierarchical spatial features. In the improved BiFPN structure, the add operation is replaced with a concatenate operation at the channel level, connecting the eigenvectors directly and summing the input eigenvector dimensions. Then, the number of channels is unified through a 1×1 convolutional layer to obtain as much feature information as possible. Before entering the feature fusion module, 1×1 convolution is applied to unify the output channel number of 6th layer to 512, which reduces the computation and retains more efficient feature information at the same time. The flow diagram of SSD algorithm is shown in Algorithm 1.
**Algorithm 1** SSD feature extraction.**Require:** Input image sequences $I(I_{1},I_{2},...,I_{n})$;
   **for**
$I_{x}$ in *I*
**do**      Generate feature map $F(f_{1},f_{2},...,f_{m})$ through CNN feature extraction;      **for**
conv in (conv4−3,conv7,conv8−2,conv9−2,conv10−2,conv11−2)
**do**         Extract feature map $\left(f_{conv4-3},...,f_{conv11-2}\right)$;         Each layer of feature maps obtain more efficient feature information through the attention mechanism;         
Use the improved BiFPN to fuse the new feature maps;         Construct different bounding boxes in size $S(s_{1},...,s_{k})$;**      end for**      Input NMS algorithm;      Output default box after selection;**   end for**   Algorithm stops.**Ensure:** Bounding boxes and classes of objects.

### 3.2. More Efficient Activation Functions

Mish activation function, a deep learning activation function proposed by Diganta Misra [33], is adopted in this paper. The Mish activation function is a stationary and non-mono activation function. The gradient of the Mish function is smoother than ReLU, and the smooth activation function allows more information to penetrate into the neural network, so as to obtain better accuracy and generalization. The Mish function is approaching in direct proportion on the positive x axis, and approaching zero on the negative x axis. The upper boundary-less property of Mish function avoids the saturation problem caused by the cap. In theory, a slight allowance for negative values also enables it to obtain a smoother gradient flow, rather than a hard zero boundary like the ReLU function.

Equation (1) shows the Mish function. The accuracy of the modified network can be significantly improved by using the Mish function.

(1) $$\mathrm{Mish}\left(x\right)=x\times \tanh(\ln\left(1+e^{x}\right))$$

where *x* denotes the activated variable.

### 3.3. Attention Mechanism

In order to make full use of the extracted features, the global context attention mechanism of GCNet [34] is integrated before the feature fusion module, so that the effective feature information has higher weight.

**Figure 2:**
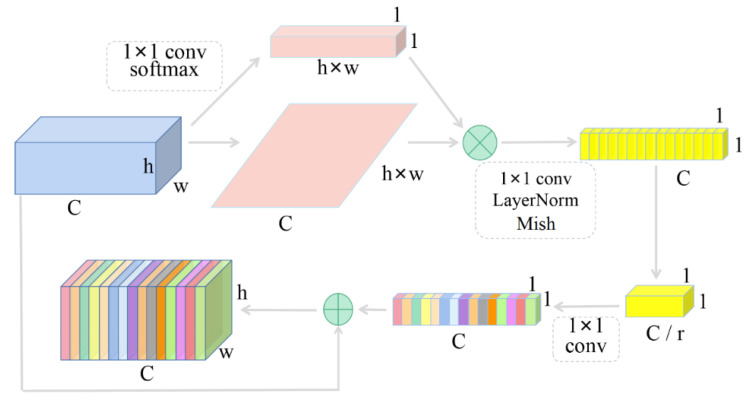
Global context attention mechanism.

As shown in Figure 2, the global average pooling module is used to model the global context of the feature graph to capture the long distance dependencies. After that, two convolutional layers are used to stimulate the global context and capture the dependency relationship between channels before the LN layer is added. In addition to the lightweight model, it is also used as a regular term to improve the model generalization. Mish is also used as the activation function. Finally, the broadcast Element-wise addition is used to calibrate the channel feature weights. The global self-attention mechanism introduced in this paper significantly improves the performance of the model with only a small increase in computational cost.

## 4. Road Scene Modeling

The traffic elements can be located through the object detection framework mentioned above. Based on those detection results, an unsupervised CycleGAN is applied to inpaint the foreground area of the image, so as to obtain image sequences of high quality. Aiming at the characteristics of image sequences, the foreground region inpainting algorithm proposed in this paper is divided into two stages: optical flow inpainting and content inpainting. In the stage of optical flow inpainting, the optical flow map is generated by using the changes of pixels in the image sequence in the time domain. The optical flow map consists of two channels $\left(v_{x},v_{y}\right)$, where $\left(v_{x},v_{y}\right)$ represents the displacement of each pixel in the image in the x direction and y direction, and the unsupervised CycleGAN is applied to inpaint the optical flow map of the two channels. The two channels are concatenated into a complete optical flow map, and then the inpainted optical flow map is applied to track the target area corresponding to the next frame. In the stage of content inpainting, for each pixel coordinate $\hat{x}$ on the hole area of the image to be restored, we track the pixel value of the corresponding coordinate of its adjacent k frames of image, and then use the statistical information of the pixel trajectory to establish a Gaussian mixture model(GMM). The parameters of the model will be updated according to the order of the trajectory points, and the mean value of the last Gaussian distribution model will be adopted as the pixel value of the coordinate $\hat{x}$ of the cavity area of the image to be inpainted. This method can efficiently use the pixel statistical characteristics of adjacent k frames of images to adaptively fill in the pixel values of the target background holes. Finally, the road scene and traffic elements are modeled with the image sequence as input. This section mainly describes the optical flow inpainting method based on CycleGAN. The image inpainting using Gaussian mixture models (GMM) is then introduced based on the optical flow inpainting results. Finally, the road scene models are constructed.

### 4.1. Optical Flow Inpainting Based on CycleGAN

CycleGAN is an unsupervised generative adversarial network. The main idea is to train two pairs of generator-discriminator models to convert images from one domain to another, inspired by dual cycle consistency. CycleGAN can capture special characteristics of one image collection and then figures out how these characteristics could be translated into the other image collection, all in the absence of any paired training examples. Therefore, we utilize CycleGAN to convert optical flow from damaged region to inpainting region.

**Figure 3:**
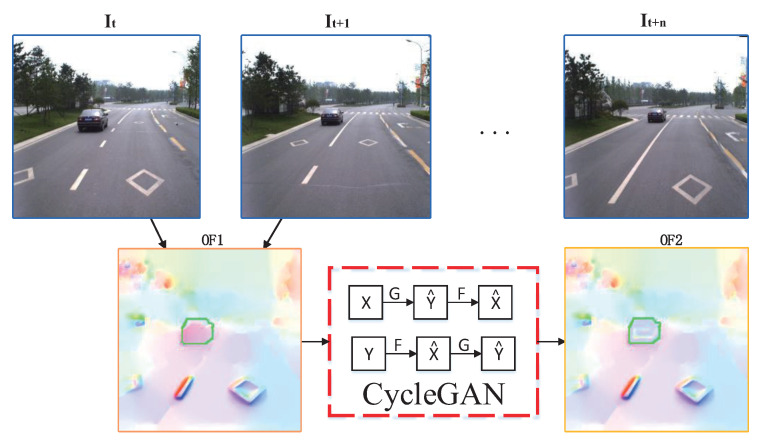
Flow diagram of optical flow inpainting.

Foreground regions like vehicles are regard as missing pixels, and optical flow is inpainted using CycleGAN, as shown in Figure 3. First, contours of the foreground object are tracked for each frame of the image sequence, and optical flow OF1 is generated by adjacent frames $I_{t}$ and $I_{t+1}$. Second, the tracked foreground region in optical flow OF1 is repaired using CycleGAN, and inpainting optical flow OF2 is acquired. As the optical flow information between foreground region and background region is significantly different, the background flow region can be applied to predict and inpaint foreground region. Finally, the missing region of image $I_{t}$ can be inpainted by the background region of image $I_{t+1}$. In the same way, we can obtain *n* corresponding background region by generating and inpainting optical flow of images $I_{t}$ and $I_{t+i}$(1≤i≤n).

In our method, the optical flow inpainting is implemented using an unsupervised CycleGAN. Optical flow includes $\left(v_{x};v_{y}\right)$ channels. The method inpaints two channels of optical flow, respectively, and then concatenates the repaired two channels into complete optical flow.

**Figure 4:**
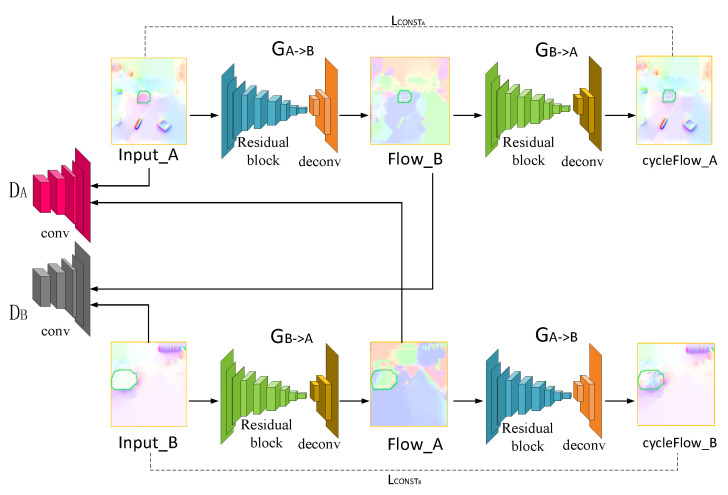
The network structure of CycleGAN.

The network structure of CycleGAN includes two generators $G_{A->B}$ and $G_{B->A}$, as well as two discriminators $D_{A}$ and $D_{B}$, as shown in Figure 4. Our goal is to learn mapping functions including $G_{A->B}$: optical flow with damaged region −> inpainting optical flow, and $G_{B->A}$: inpainting optical flow −> optical flow with damaged region. The generator consists of 9 residual blocks and 3 deconvolution layers, which are used to generate optical flow. The two discriminators, $D_{A}$ and $D_{B}$, consist of 5 convolutional layers. $D_{A}$ aims to distinguish between optical flow Input_A and translated optical flow Flow_A; in the same way, $D_{B}$ aims to distinguish between optical flow Input B and translated optical flow Flow_B. Our objective contains losses of generators and discriminators. We express the loss of generators as

(2) $$\begin{array}{cc} L_{G_{A}->B} & =L_{GAN_{B}}+L_{CONST_{A}}\\ \; & =\sum_{i=1}^{n}\log D_{B}\left(G_{A->B}\left({\mathrm{Input}}_{-}A\right)\right)+d\left({\mathrm{Input}}_{-}A,G_{B->A}\circ G_{A->B}\left({\mathrm{Input}}_{-}A\right)\right) \end{array}$$

(3) $$d\left(a,b\right)={\lambda ∥a-b∥}_{1}$$

where *n* denotes the number of optical flow in training set. The generator loss includes adversarial loss LGANB and cycle consistency loss LCONSTA. The generator tries to produce images that are similar to images from another domain, while the discriminator aims to distinguish between translated samples and real samples. Adversarial loss makes the generated image similar to the image of another domain by minimizing this objective against an adversary discriminator that tries to maximize it. $G_{A->B}$ (Input_A) denotes optical flow Flow_B. The cycle consistency loss can be observed that the reconstructed images end up matching closely to the input images. $G_{B->A}\circ G_{A->B}$ (Input_A) denotes optical flow cycle Flow_A. λ denotes a similar parameter.

The loss function of the discriminator is defined as follows,

(4) $$L_{D_{A}}=-\sum_{i=1}^{n}\log D_{A}\left({\mathrm{Input}}_{-}A\right)-\sum_{i=1}^{n}\log\left(1-D_{A}\left(G_{B->A}\left({\mathrm{Input}}_{-}B\right)\right)\right)$$

where $G_{B->A}$ (Input_B) denotes optical flow Flow_A.

The total loss is defined as follows,

(5) $$\begin{array}{c} L_{G}=L_{G_{A->B}}+L_{G_{B->A}} \end{array}$$

(6) $$\begin{array}{c} L_{D}=L_{D_{A}}+L_{D_{B}} \end{array}$$

As CycleGAN does not require the training data in pairs, we utilize the optical flow generated by image sequences with foreground objects such as vehicles and pedestrians as the damaged region dataset, and utilize the optical flow generated by image sequences without foreground objects as the inpainting dataset. We shuffle the order of the dataset before each training epoch and set the learning rate to 0.00002. After the alternation training between generators and discriminators, we obtain the network finally which can inpaint optical flow.

### 4.2. Inpainting of Image Sequences Based on GMM

The image content is inpainted by *n* corresponding background region which is generated from inpainting optical flow result of images $I_{t}$ and $I_{t+i}$(1≤i≤n). To obtain a pure background image from a sequence of images, we adopt a pixel-based adaptive method based on a Gaussian mixture model (GMM).

At this time, we use the GMM to predict the most likely color information of each pixel in the missing region, so as to implement the image content inpainting.

For each pixel $\hat{x}$ in the missing region, we trace its history:

(7) $$\begin{array}{c} \left\{t_{i}:t_{i}=I_{T}\left(\hat{x},i\right),1\leq i\leq n\right\} \end{array}$$

where $I_{T}\left(\hat{x};i\right)$ denotes the pixel value of $i_{th}$ frame. Specifically, $t_{0}$ is defined as the pixel value $\hat{x}$ to be inpainted.

A mixture of *N* Gaussian distributions is defined:

(8) $$\begin{array}{c} x_{i}∼\sum_{j=1}^{N}w_{i,j}N\left(\mu_{i,j},\sigma_{i,j}^{2}\right),0\leq i\leq k \end{array}$$

where $x_{i}$ denotes the pixel value after *i*th update. *N* denotes the number of Gaussian distributions with a mean value $\mu_{i,j}$ and variance $\sigma_{i,j}^{2}$. $w_{i,j}$ denotes the weight of the *j*th Gaussian distributions in *i*th update.

The parameters of the model are sequentially updated by checking against the existing *N* Gaussian distributions to find if a match occurs. If two or more matches occur, only the best matched distribution is chosen by comparing the Euler distance of the mean value $\mu_{i,j}$ and the current pixel value. If none of the *N* distributions match the current pixel value, the least probable distribution is replaced with a distribution with the current value as its mean.

Whether the *i*th distribution matched or not, the weight needs to update:

(9) $$\begin{array}{c} w_{i,j}=\left(1-\gamma \right)w_{i-1,j}+\gamma \varepsilon_{i-1,j} \end{array}$$

where γ denotes the learning rate. $\varepsilon_{i-1,j}$ is a positive parameter as the matched distribution or zero for remaining distributions.

The mean and variance of the matched distributions are computed as follows,

(10) $$\begin{array}{c} \mu_{i,j}=\left(1-\gamma \right)\mu_{i,j}+\gamma t_{i-1} \end{array}$$

(11) $$\begin{array}{c} \sigma_{i,j}^{2}=\min\left(\sigma_{\min}^{2},\left(1-\gamma \right)\sigma_{i-1,j}^{2}+\gamma ||t_{i-1}-\mu_{i-1,j}{∥}^{2}\right) \end{array}$$

where $\sigma_{\min}^{2}$ denotes the minimum threshold of the variance. $t_{i-1}$ denotes the current pixel value, which is used to avoid the problem that the variance is too small to be successfully matched.

After *n* iterations of parameter update using each image frame, $\varphi_{j}=w_{k,j}/\sigma_{k,j}$ for each Gaussian model is computed. Due to the largest $\varphi_{j}$ means the distribution has the most supporting evidence and the least variance, we choose the mean of the Gaussian model with the largest $\varphi_{j}$ as the pixel value of t0. In the same way, we inpaint each pixel of the damaged region, and a pure background image was obtained. Thus, complete background image sequences are obtained when this update procedure is done for all the pixels and all the frames.

Finally, the mean of the distribution is taken as the pixel value of the empty region of the image to be repaired at $\hat{x}$. The algorithm flow is shown in Algorithm 2.
**Algorithm 2** Inpainting of image sequences based on Gaussian mixture model.**Require:** Inpainting optical flow results of images $I_{t}$ and $I_{t+i}\left(1\leq i\leq n\right)$;   **for** pixel $\hat{x_{i}}$ in missing region **do**      **for**
$N_{j}$ in *N* Gaussian models **do**         Compute mixture of $N_{j}$ Gaussiasn distrubutions;         update weight: $\omega_{i,j}=\left(1-\gamma \right)\omega_{i-1,j}+\gamma_{\epsilon i-1,j}$;      **end for**      Choose the mean of Gaussian model $\omega_{k},j{/}_{k},j$ as pixel value;   **end for**   Algorithm stops.**Ensure:** Impainted sequences $\hat{I_{t}},\hat{I_{t+i}}$.

### 4.3. Road Scene Construction and Simulation

After the image inpainting procedure, the pure background image can be obtained. The scene models are then constructed based on the scene stages. These form the basis of the road scene simulation process. A scene graph model can be defined for each image: G=(V,E), where *V* denotes the traffic elements and *E* represents their relationships. The “floor-wall” traffic scenes can be constructed based on the scene models, where the road plane is considered to be the horizontal plane, and the background walls and traffic elements are assumed to stand vertically to the road plane.

Scene modeling can be applied to the simulation of three-dimensional traffic scenes to simulate the driving modes of vehicles under different scene conditions. By constructing a scene model with the floor wall structure, the user can adjust the viewpoint of observation by entering instructions and switch to a bird’s eye view mode or roaming mode. The former can freely set up the virtual road environment of the scene and add vehicles, obstacles, etc. The latter can generate new viewpoints according to the movement, and users can switch to different perspectives by moving the scene model.

## 5. Experiments and Comparisons

The experiments and comparisons of the proposed framework mainly consist of two parts: (1) Object detection experiment and (2) road scene modeling experiment.

### 5.1. Object Detection Experiment

In this section, we first introduce the datasets utilized to evaluate the object detection model, and then conduct experiments and specific comparative analysis on the selected models on each data set.

#### 5.1.1. Datasets and Metrics

The proposed methods are verified on VOC2007, UA-DETRAC, and the TSD-max dataset of Xi’an Jiaotong University. The UA-DETRAC dataset contains 100 video clips with a total of 138,252 images, and four categories of car, van, bus, and others, and manually labeled 8250 vehicles and 1.21 million target object boxes. The VOC2007 data set contains the training set (5011) and the test set (4952) including 9963 images in 20 categories. In the UA-DETRAC data set, the training set contains 60 video sequences, and the test set contains 40 different sequences.

In order to evaluate the performance of the proposed model on the validation set, we used mean average precision (mAP) and frame per second (FPS) as indicators to compare with other object detection models.

#### 5.1.2. Object Detection Experiments

The proposed model was pre-trained on the MSCOCO (2014) data set, 100 epochs were iterated, and the initial learning rate was set to 0.01.

First of all, we fine-tune each model on VOC2007, UA-DETRAC and the TSD-max dataset of Xi’an Jiaotong University, respectively, and compare the proposed model with the existing model. The evaluation metrics are as follows.

- mAP: The average accuracy under different categories. The experimental results prove that the BiSSD model proposed in this paper has the highest mAP in the experiments of the three data sets. It further demonstrates the effectiveness of asymmetric convolution and global context attention mechanism.
- FPS: In intelligent transportation systems, the real-time detection speed of the model is very important. The proposed model uses the GCNet attention framework and a large number of small convolution kernels, which greatly reduces the computation. The result in Table 1 and Table 2 demonstrate that the proposed method shows a strong real-time advantage.

**Figure 5:**
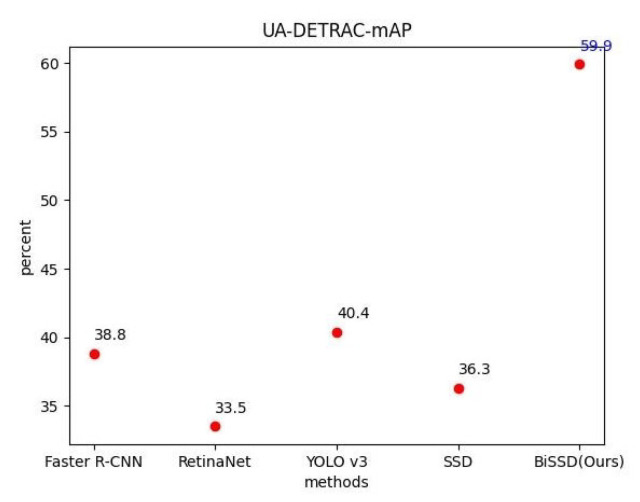
MAP index comparison of UA-DETRAC data set test.

Table 1 shows that the model proposed in this paper has different detection effects for different categories of targets. Compared with other methods, there are no significant difference in the detection effect of vehicle and road categories. The proposed method shows advantages in both mAP and FPS metrics compared with the existing Faster R-CNN, RetinaNet, YOLO V3, and SSD methods for motorcycle and pedestrian categories. On the basis of SSD feature extraction, our method added improved BiFPN feature fusion, effectively retained the fine-grained information, and significantly improved the detection effect for the target with small size. Table 2 and Figure 5 show the test results based on the UA-DETRAC data set. The average accuracy in this paper is significantly improved compared with other methods. Meanwhile, the real-time detection speed index of the model is ensured to be at a high level.

**Figure 6:**
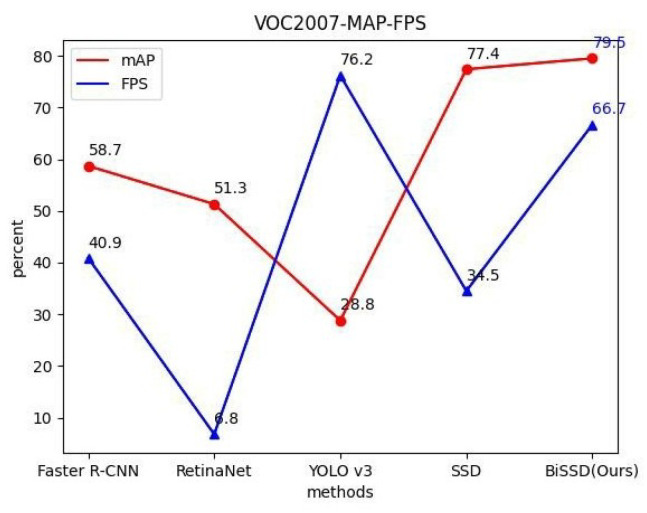
MAP index comparison of VOC2007 data set test.

The comparison between the BiSSD method in this paper and the experimental indicators of other models on VOC2007 dataset is shown in Figure 6. Both the mAP and FPS indexes are at a high level. Although the YOLO V3 algorithm ensures a high real-time detection rate, it is at the expense of accuracy. Our BiSSD method balances both of them, providing the highest accuracy compared to other models and minimizing the loss of detection speed.

**Figure 7:**
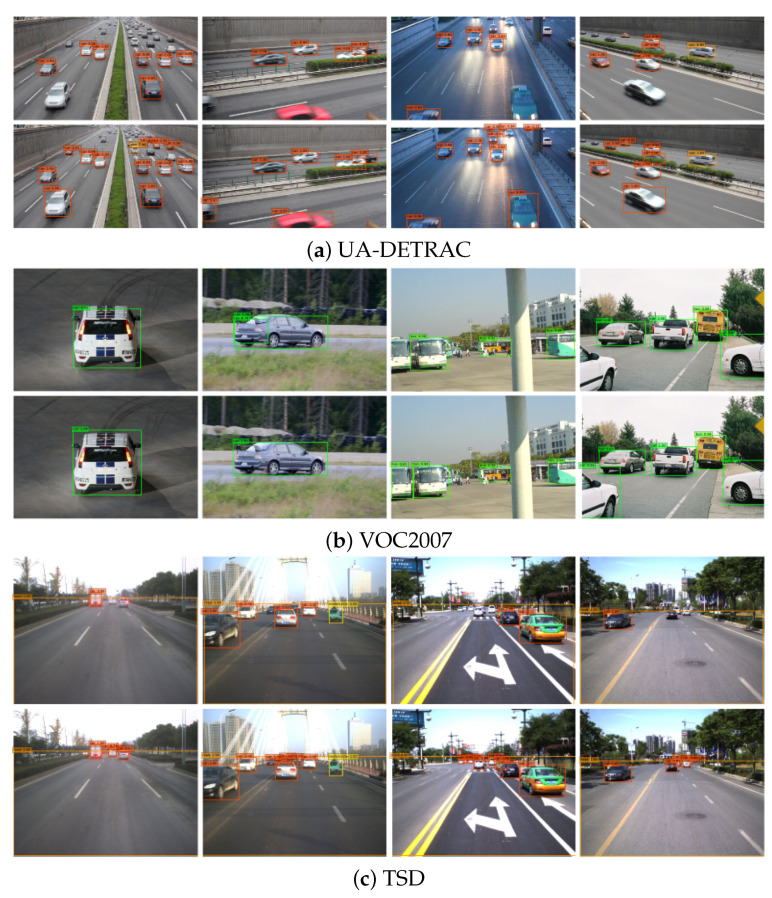
Qualitative comparative test.

Meanwhile, we conducted qualitative experiments to verify the effectiveness of the proposed model. Figure 7 shows the qualitative experimental results on UA-DETRAC (Figure 7a), VOC2007 (Figure 7b), and TSD (Figure 7c) data sets. The lower part of the comparison graphs is the test result of our method, while the upper part is the original SSD test result. It can be seen that the detection effect of our improved model is better than the original SSD in terms of confidence, target frame regression accuracy, and detection accuracy.

### 5.2. Road Scene Modeling Experiment

#### 5.2.1. Image Inpainting

The experiments and comparisons are conducted on a computer with NVIDIA Geforce 1080Ti GPUs of 11G memory. We evaluate the proposed method from both quantitative and qualitative aspects. The experiments and comparisons are based on the TSD-max [36] dataset (Xi ’an Jiaotong University).

**Figure 8:**
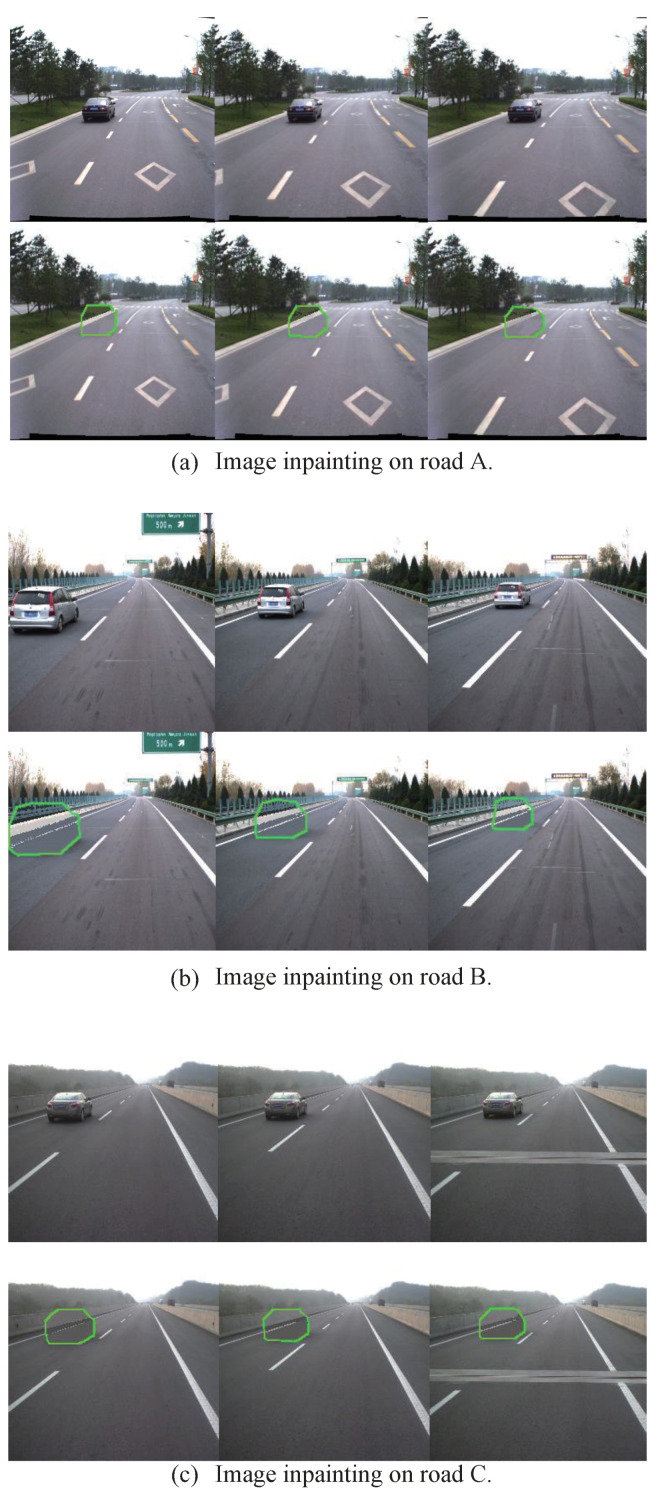
Qualitative results for image inpainting on TSD-max.

We divide the testing set into nine groups; the image sequence of each group has 50 frames with 256 × 256 size. In order to take both visual and semantic coherence into account, we conduct qualitative comparisons on the former three groups, as shown in Figure 8. Our method is able to generate semantically-reasonable and visually realistic results with clear textures and consistent structures with context.

**Figure 9:**
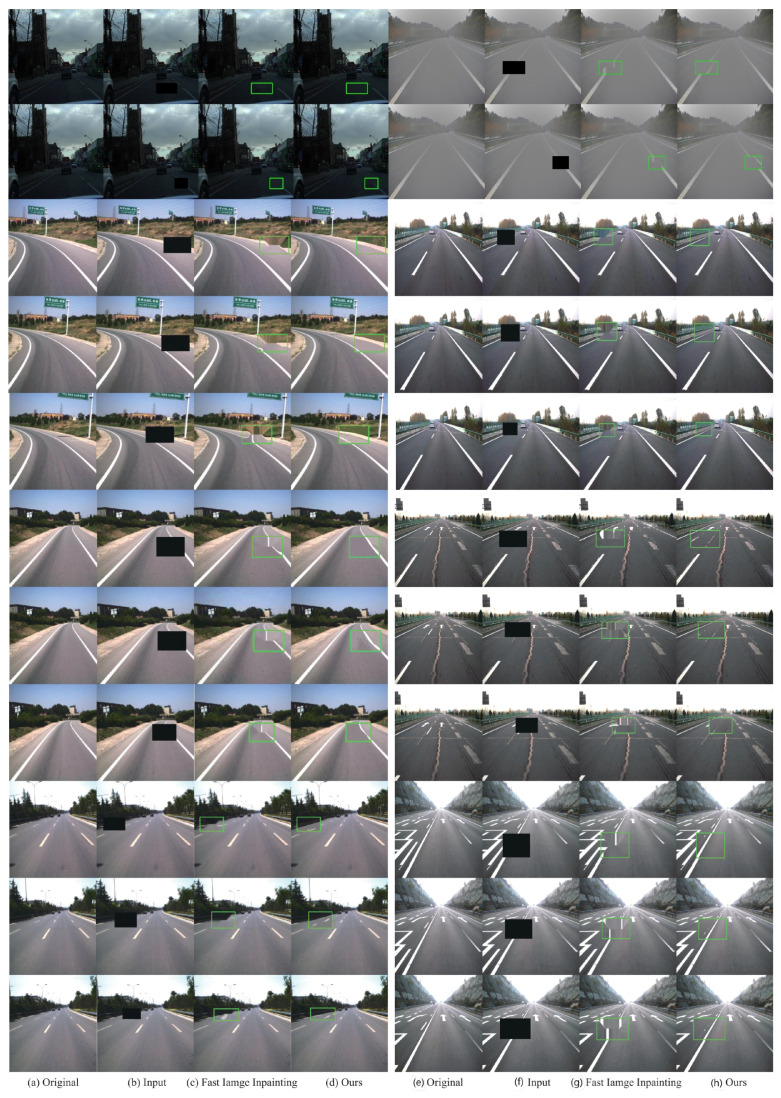
Quantitative results for image inpainting with black masks on TSD-max.

Quantitative comparisons are conducted in the last six groups. As shown in Figure 9, in each pair, Figure 9a,e is the original image, and Figure 9b,f is a damaged image masked in black. Figure 9c,d,g,h shows the results of fast image inpainting and our proposed method, respectively. All images are randomly masked for testing, and we use peak signal-to-noise ratio (PSNR) [10] and structural similarity index (SSIM) [11] as experimental metrics. The comparable performance of the proposed approach against the fast image inpainting method [12]. Concerning our proposed method, the best setting yields results in 39.27 dB/0.98 in terms of PSNR and SSIM. And compared with [12], our method is 3.92 dB/0.05 in terms of PSNR and SSIM higher on average.

**Figure 10:**
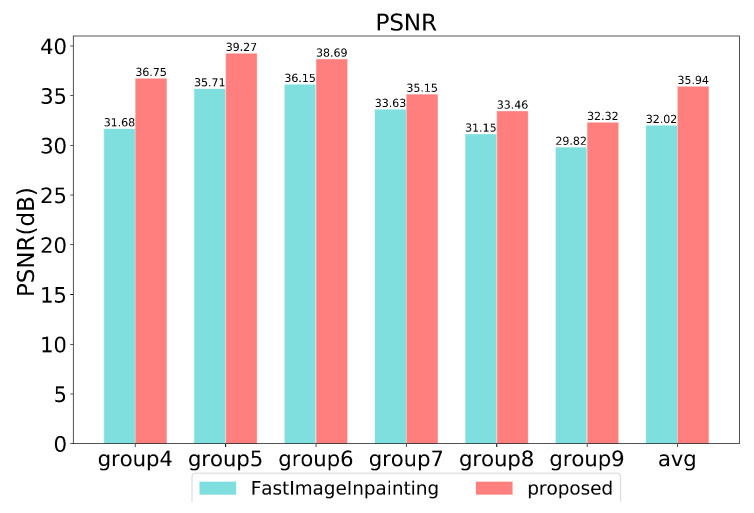
Quantitative results in terms of PSNR on TSD-max.

**Figure 11:**
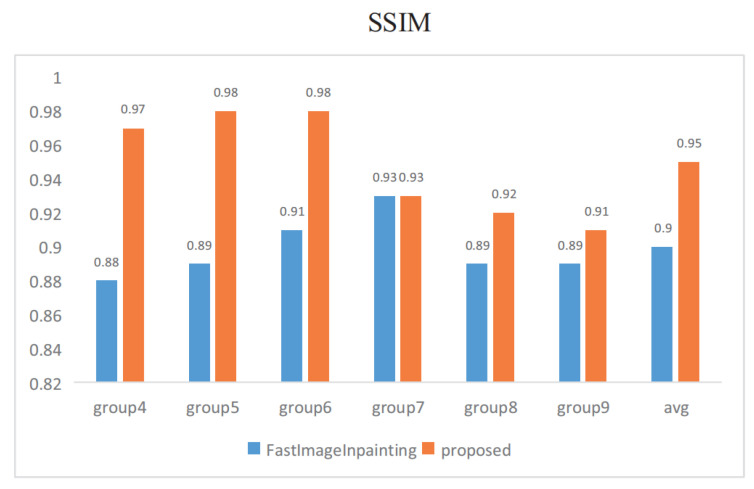
Quantitative results in terms of SSIM on TSD-max.

The image inpainting speed is evaluated for different stages in Figure 10 and Figure 11. Stage1 spends 0.98 s on average while stage2 spends 1.22 s on average. It takes more time for image inpainting using GMM than optical flow inpainting using CycleGAN.

#### 5.2.2. Road Scene Models Construction

**Figure 12:**
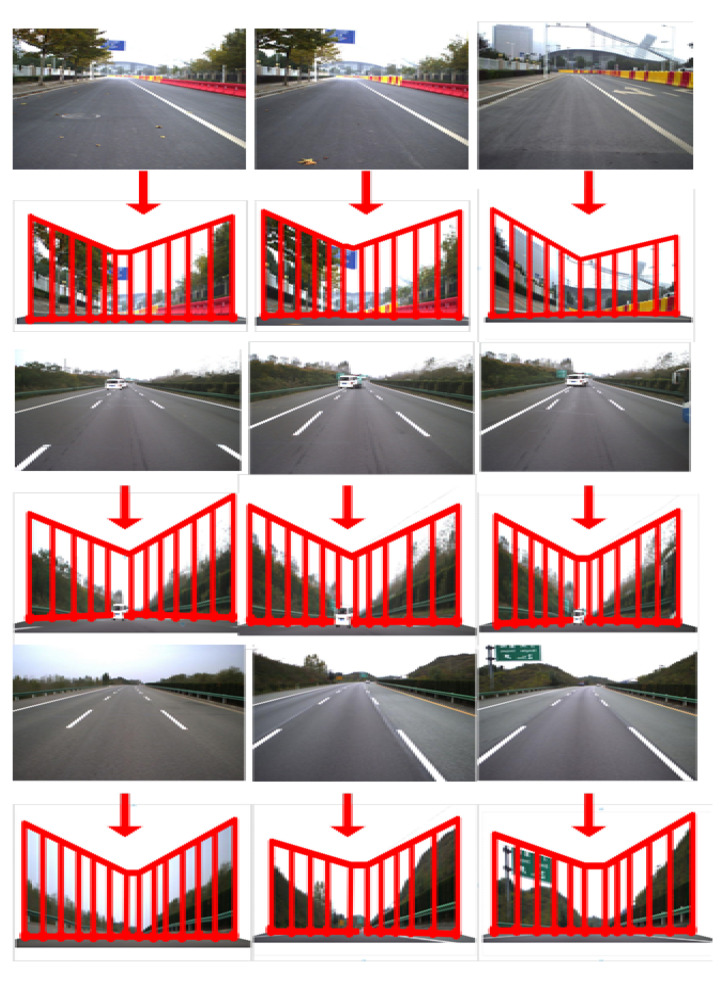
Traffic scenes construction from road images.

Based on the results of traffic elements detection and road image inpainting, the road scene models can be constructed. The experiments are based on the TSD-max dataset [36], which is constructed by institute of Artificial Intelligence and Robotics, Xi’an Jiaotong University. We use the proposed semantic segmentation network of the encoder–decoder architecture with channel modules and depth separable modules to semantically divide the road image, and then build a roaming background based on the Spatio-temporal graph model, and add the foreground and supplementary objects to the establishment. In a good background model, traffic incident simulation can be realized. We conducted experiments on 100 different traffic scenes. The average modeling time for each image is 0.029 s. Thirty-four monocular images can be modeled per second, which can meet the real-time requirements of traffic scene modeling. This method can design and simulate common traffic scenarios, and then realize the offline simulation test of unmanned vehicles. The background models of the road scenes are constructed according to a 3D corridor structure. The foreground traffic elements are assumed to stand perpendicularly to the road plane of the background models, as shown in Figure 12.

## 6. Conclusions and Future Works

In this paper, a novel framework for the analysis and synthesis of traffic elements is proposed to construct road scene models from road image sequences. For the traffic object detection model, we combined the SSD feature extraction and BiFPN feature fusion algorithm, having achieved satisfactory detection results, especially in the detection of small targets. Furthermore, we improved BiFPN feature fusion and used an attention mechanism to detect traffic elements. After that, we inpainted the image sequence based on the detection result and sent the restored image to the road scene construction model. The “floor-wall” traffic scenes then can be constructed based on those scene models. Experimental results demonstrate the effectiveness of the proposed method, and the result of scene modeling can provide users with a virtual driving experience from multiple perspectives.

In our future research, the model of feature extraction and fusion will be refined. The accuracy and speed for small object detection will be improved. Furthermore, the application of traffic scene construction will be developed, which combines the traffic elements detection and image inpainting to construct better scene models. 

## Figures and Tables

**Table 1:** Experimental results of each model on the large data set.

	AP				mAP	FPS
	Car	Motorcycle	Person	Road	%	f/s
Faster R-CNN [28]	47.6	36.1	18.6	90.9	48.3	40.9
RetinaNet [35]	40.2	35.3	20.1	99.4	48.7	5.4
YOLO v3 [29]	56.7	29.2	9.4	99.7	48.8	83.3
SSD [6]	46.7	54.1	17.3	91.0	52.3	47.6
BiSSD(Ours)	46.0	54.6	22.9	90.9	53.6	76.9

**Table 2:** Experimental results of each model on UA-DETRAC data set.

	AP				mAP	FPS
	Car	Van	Track	Others	%	f/s
Faster R-CNN [28]	61.2	40.2	50.7	2.9	38.8	34.5
RetinaNet [35]	67.0	11.3	52.4	3.1	33.5	1.8
YOLO v3 [29]	54.4	20.1	50.4	8.2	40.4	51.5
SSD [6]	58.5	38.8	33.6	14.1	36.3	14.3
BiSSD(Ours)	68.1	47.0	75.0	49.7	59.9	50.0
